# Study on the Mechanism of *Periplaneta americana* Extract to Accelerate Wound Healing after Diabetic Anal Fistula Operation Based on Network Pharmacology

**DOI:** 10.1155/2021/6659154

**Published:** 2021-03-09

**Authors:** Fengfei Wang, Shuai Li, Le Ma, Yuefei Geng, Yongmei Shen, Juanni Zeng

**Affiliations:** ^1^The Second Hospital of Hunan University of Traditional Chinese Medicine, Hunan, Changsha 410005, China; ^2^Hunan University of Traditional Chinese Medicine, Hunan, Changsha 410208, China; ^3^The First Hospital of Hunan University of Traditional Chinese Medicine, Hunan, Changsha 410007, China; ^4^Sichuan Key Laboratory of Medical American Cockroach, Sichuan, Chengdu 610000, China

## Abstract

**Objective:**

Using network pharmacology research methods to explore the healing mechanism of American cockroach extract to accelerate wound healing after diabetic anal fistula surgery.

**Method:**

The main chemical constituents of extracts from *Periplaneta americana* were collected by literature retrieval. Chemical composition and targets related to diabetic anal fistula wound could be predicted based on PubChem, Swiss Target Prediction, OMIM, and GeneCards databases, and the putative targets of *Periplaneta americana* extraction (PAE) for diabetic anal fistula wound were obtained by Venn diagram. These common targets were predicted using the String database for protein-protein interaction (PPI) network and then screening key genes through Cytohubba. Meanwhile, the above targets were analyzed using the DAVID database for gene ontology (GO) enrichment analyses and the Kyoto Encyclopedia of Genes and Genomes (KEGG) path enrichment analyses.

**Results:**

A total of 12 chemical components of PAE were obtained by literature retrieval, and 61 therapeutic targets that may accelerate the healing of diabetic anal fistula wounds were predicted by the database. According to PPI network analysis, PAE accelerates wound healing after diabetic anal fistula surgery which may be related to proteins such as AKT1, VEGFA, EGFR, CASP3, STAT3, MAPK1, TNF, JUN, ESR1, and MMP9. GO analysis results show that targets of PAE to promote wound healing were mainly involved in biological processes such as cell proliferation, macrophage differentiation, angiogenesis, and response to hypoxia. KEGG analysis showed that the target genes were mainly concentrated in the PI3K-Akt signaling pathway, HIF-1 signaling pathway, and estrogen signaling pathway.

**Conclusion:**

*Periplaneta americana* extract regulates multiple targets and multiple pathways to promote wound healing after diabetic anal fistula surgery. PI3K-Akt signaling pathway, HIF-1 signaling pathway, and sex hormone signaling pathway may be key pathways in the process of *Periplaneta americana* extract promoting wound healing.

## 1. Introduction

Anal fistula is a tube located between the perianal skin and the rectum. It is caused by chronic infection and epithelialization of the drainage tube [1]. It is one of the common diseases in the anorectal department, and surgery is the first choice. Epidemiological data show that the total number of diabetes patients in the world was 463 million in 2019, and it is estimated that it will rise to 578 million in 2030 and 700 million in 2045 [2]. Prolonged generation of pathological blood sugar elevation in the body can lead to the sequel of advanced glycation end products in the body, which provide a good nutritional environment for the growth of bacteria, and easily cause wound tissue infection and necrosis, and hinder wound healing. Diabetes is a risk factor leading to slow wound healing after anal fistula surgery [3–5].

In wound healing, some herbal and animal Chinese medicines have very good clinical effects [6–8], and Quyushengxin formula can promote mucosal healing in UC patients [9]. In traditional Persian medicine [10], the plants' hard tissues such as roots or barks were boiled in water. Subsequently, the resulting extract was boiled in combination with sesame or olive oil until its water part was lost to exert its efficacy. *Periplaneta americana* (PA), the American cockroach, has the largest body size in the family Blattidae. It is an animal traditional Chinese medicine. Physiological and pharmacological studies have demonstrated that PA constituents have favorable tissue-repairing, antibacterial, antitumor, and immunity-enhancing activities [11]. Additionally, this insect has been widely used for the treatment of various wounds, ulcers, fistulas, bedsores, and burns [12]. Consequently, the formulation of many TCM preparations, among them, Kangfuxin (*Periplaneta americana* extract, PAE), is a liquid preparation that includes ingredients from PA that has been used to treat different skin or mucosa injuries in China for more than 40 years. Our team applied PAE to wounds after anorectal diseases in the early years and found that the effect of promoting wound healing was very good [13]. Besides, it has been reported that PAE can promote postoperative wound healing in diabetic patients [14], but there are few studies on postoperative wounds in diabetes patients with anal fistula. We hypothesize that PAE can promote postoperative wound healing in diabetes patients with anal fistula, and its mechanism for promoting healing remains to be further studied.

Network pharmacology is an emerging research method first proposed by Andrew L Hopkins in Nature Biotechnology in 2007 [15]. It is based on drug databases, disease databases, gene pleiotropy, computational biology, and network biology analysis, etc., to construct a “drug-target-disease‐pathway” interaction network and to comprehensively analyze the relationship of drug components, genes, and diseases, thereby revealing the intervention mechanism of drugs on the disease. Its holistic and dynamic characteristics, the principle of multitarget, and multichannel administration are highly consistent with the holistic view of traditional Chinese medicine and the treatment principle of syndrome differentiation. The purpose of this study is to use the research methods of network pharmacology to initially find the target of PAE in repairing postoperative wounds of diabetes patients with anal fistula and to provide a theoretical basis for further application.

## 2. Materials and Methods

### 2.1. Effective Chemical Ingredient Screening

There are rarely studies on the chemical components of *Periplaneta americana*, and no relevant chemical ingredients are included in the TCMSP database (http://ibts.hkbu.edu.hk/LSP/Tcmsp.php). We use “American Cockroach (*Periplaneta americana*),” “KANG FU XIN YE,” as the keyword to retrieve in the PubMed and CNKI databases. 12 compounds related to wound healing were screened out through literature research [11, 12, 16]: cytosine, cytidine, thymine, uracil, guanosine, adenine, hypoxanthine, inosine, uridine, cyclo-(L-Val-L-Pro), arbutin, and (E)-3-hexenyl-*β*-D-glucopyranoside.

### 2.2. Target Prediction of Drug

The compound was imported into PubChem (https://pubchem.ncbi.nlm.nih.gov/) to obtain SMILES, and the Swiss Target Prediction database (http://www.swisstargetprediction.ch/) was used to predict potential targets and limit the species: Homo sapiens. The target point of the drug component was obtained.

### 2.3. Drug-Disease-Related Target Screening

Disease targets are derived from the Human Mendelian Genetic Database (OMIM) (http://www.omim.org/) and the human genome annotation database (GeneCards) (https://www.genecards.org/) with the keywords “diabetic anal fistula wound.” Then, the genes of the two databases were integrated to remove the duplicates to obtain the relevant targets of diabetic anal fistula wounds. Finally, the Venn diagram was used to find out the intersection genes of disease target genes and drug target genes, that is, the potential target of American cockroach extract in treating diabetic anal fistula wounds.

### 2.4. Network Construction and Analyses

#### 2.4.1. Construction of the Compound-Target Network

The drug components of PAE and their potential targets for promoting wound healing after diabetic anal fistula surgery were introduced into Cytoscape 3.1.1 to construct a network diagram of active ingredients and disease targets. The network was analyzed through the network analysis function. The degree, closeness, and betweenness are important parameters for evaluating active ingredients and targets.

#### 2.4.2. Construction of Protein-Protein Interaction Network

The intersection gene was uploaded to the String database (https://string-db.org/), the species were limited to “Homo sapiens,” the minimum interaction threshold was set to “medium confidence” 0.4, and nodes without network connections were hidden. The protein-protein interaction (PPI) network diagram was obtained. The Cytoscape 3.1.1 software was used to realize the visualization of the network, and then, the Cytohubba plug-in was used to screen the top 10 key genes according to degree.

#### 2.4.3. GO and KEGG Analyses

The potential targets of PAE in promoting wound healing after diabetic anal fistula surgery were analyzed by Gene Ontology enrichment and Kyoto Encyclopedia of Genes and Genomes pathway enrichment in the Database for Annotation, Visualization, and Integrated Discovery (DAVID) v6.8, *p* value <0.05, and the species selected were “Homo sapiens,” elucidating the potential healing mechanism of American cockroach extract in the treatment of diabetic anal fistula wounds.

## 3. Results

### 3.1. Targets of Active Ingredients in *Periplaneta americana*

There are a total of 797 predicted targets for 12 compounds, including 6 targets in cytosine, 100 in cytidine, 19 in thymine, 25 in uracil, 100 in guanosine, 32 in adenine, 32 targets in hypoxanthine, 100 in inosine, 100 in uridine, 100 in cyclo-(L-Val-L-Pro), 86 in arbutin targets, and 97 targets in (E)-3-hexenyl-*β*-D-glucopyranoside. The 12 compound target genes were merged, and then the duplicate genes were deleted to obtain 370 drug targets.

### 3.2. *Periplaneta americana* Treatment Targets for Diabetic Anal Fistula Wounds

**Figure 1:**
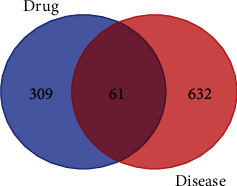
Target Venn diagram of *Periplaneta americana* and diabetic anal fistula wounds.

693 targets related to diabetic anal fistula wounds were retrieved through OMIM and GeneCards disease databases. 61 targets for the treatment of diabetic anal fistula wounds in *Periplaneta americana* were obtained by the Venn diagram online tool (Figure 1).

### 3.3. Compound-Target Network

**Figure 2:**
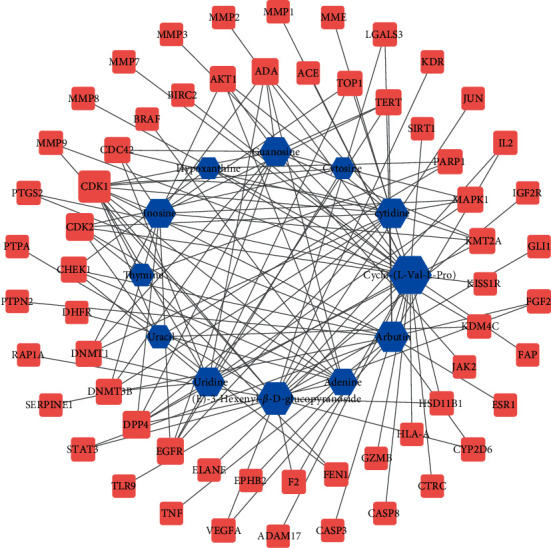
Blue represents the compound and red represents the target gene. The greater the degree value, the larger the node.

Based on 61 common target genes as targets for the treatment of diabetic anal fistula wounds, a network diagram of drug component targets was established (Figure 2). In this network diagram, nodes represent target genes and drug components, and edges represent the interaction between drug components and target genes. There are 72 nodes and 128 edges in total. The 12 active ingredients of *Periplaneta americana* act on the same or different targets. The network diagram fully reflects the multicomponent and multitarget mechanism of PAE to promote wound healing after diabetic anal fistula surgery. Through network topology analysis, the average degree of the network graph is 3.5, the average closeness is 0.3, and the average betweenness is 0.03. For 9 components, cyclo-(L-Val-L-Pro), (E)-3-hexenyl-*β*-D-glucopyranoside, arbutin, uridine, cytidine, inosine, guanosine, adenine, and uracil, the values of degree, closeness, and betweenness are all greater than the average, suggesting that these active ingredients of *Periplaneta Americana* play an important role in the treatment of diabetic anal fistula wounds. See the main parameter values in Table 1.

### 3.4. PPI Network Analysis

**Figure 3:**
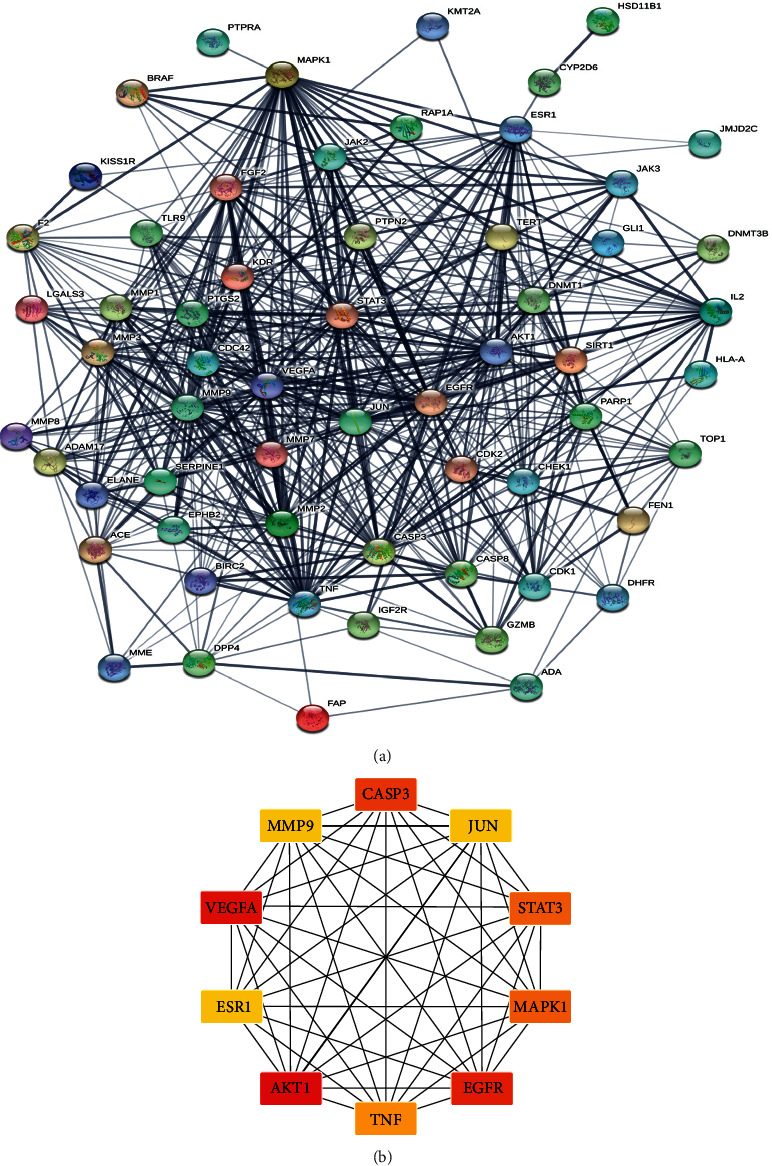
(a) Target protein interaction network (PPI). (b) The top 10 hub genes. The darker the color, the higher the score and the stronger the connection.

The target protein interaction network was analyzed by the online website STRING. Therefore, we obtained a protein interaction network of 552 interaction relationships with 60 nodes (Figure 3(a)) and calculated that the node “connectivity” of the network was 18.1 on average. By using the Cytohubba analysis tool, the top 10 hub genes were obtained according to degree, and the results show that the hub genes are AKT1, VEGFA, EGFR, CASP3, STAT3, MAPK1, TNF, JUN, ESR1, and MMP9, as shown in Figure 3(b). There is a strong interaction relationship, which may be a potential key gene for American cockroach extract to promote diabetic anal fistula wound repair.

### 3.5. Gene Ontology Enrichment Analysis

**Figure 4:**
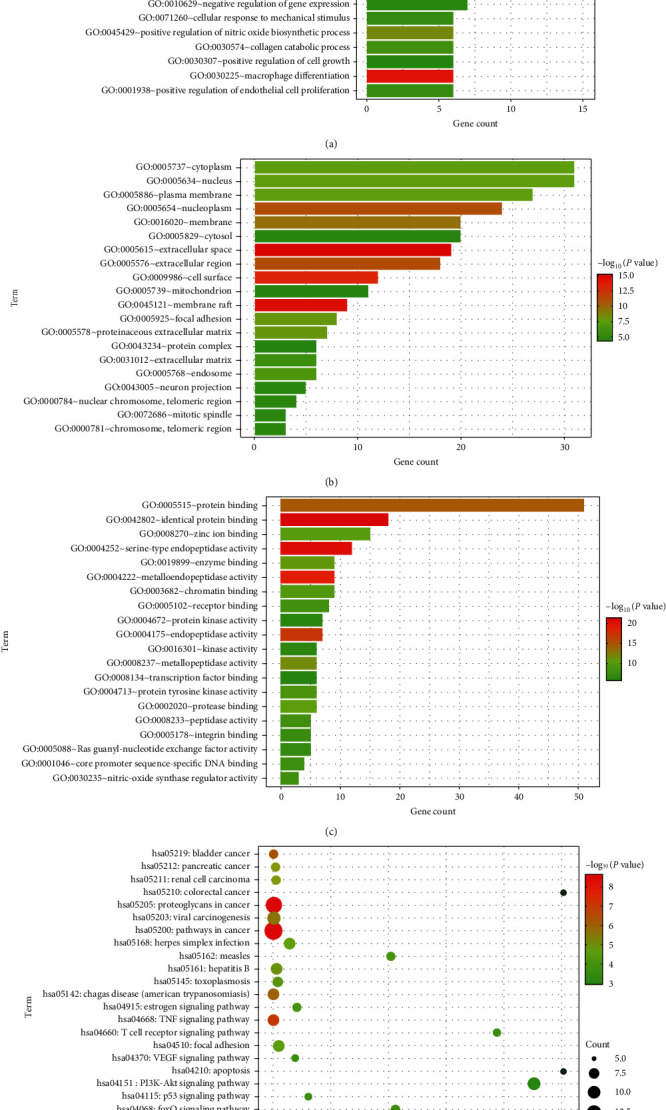
(a) Biological process, (b) cell component, (c) molecular function, and (d) KEGG pathway enrichment analysis.

We imported 61 potential target genes into the DAVID 6.8 database for GO analysis. GO analysis includes three aspects: molecular function (MF), cellular component (CC), and biological process (BP) to predict the main biological functions performed by target genes. Under the criterion of *p* < 0.05, the top 20 terms in the ranking are displayed. The results showed that BP of the target genes (Figure 4(a)) mainly occurs in the positive regulation of cell proliferation, nitric oxide biosynthesis, protein phosphorylation, and ERK1 and ERK2 cascades and shows the components involved in proteolytic metabolism, macrophage differentiation, angiogenesis, and response to hypoxia. CC (Figure 4(b)) shows the components involved in nuclear chromosomes, organelles, and plasma membranes; MF (Figure 4(c)) mainly focuses on protein and zinc ion binding, protein tyrosine kinase activity, nitric oxide synthase regulatory activity, and guanosine regulation of nucleotide exchange factor activity.

### 3.6. KEGG Pathway Enrichment Analysis

KEGG pathway analysis was performed on 61 key target genes using the DAVID 6.8 database. Under the standard of *p* < 0.05, 66 signaling pathways were screened, which were arranged in ascending order according to the *p* value. The enrichment results of the first 25 pathways showed that target genes were mainly enriched in the VEGF signaling pathway, PI3K-Akt signaling pathway, estrogen signaling pathway, MAPK signaling pathway, FoxO signaling pathway, and HIF-1 signaling pathway (Figure 4(d)). It is suggested that the above pathways may be involved in the regulation of diabetic anal fistula wound healing by *Periplaneta americana*.

## 4. Discussion

Wound healing goes through a continuous dynamic physiological process of inflammation, proliferation, and remodeling, involving a variety of cells and cytokines. Retention at any stage and failure to proceed in an orderly manner may cause delayed wound healing. In this study, a PPI network was constructed on the healing-promoting target of *Periplaneta americana* extract (PAE), and it was found that AKT1, VEGFA, EGFR, CASP3, STAT3, MAPK1, TNF, JUN, ESR1, and MMP9 interact closely. Our team's previous experimental studies have confirmed that the extract of *Periplaneta americana* maintains a high level of caspase-3 RNA expression in the early stage of wound healing, speeds up the shedding of necrotic tissue on the wound, and then slowly decreases the expression of water skin, reducing tissue hyperplasia and avoiding scar formation [17]; upregulating the expression of VEGF during the granulation phase of the wound to promote microangiogenesis in the ischemic area [18]; and downregulating the expression of Smad6 and upregulating the expression of Smad9, accelerating cell ultrastructural proliferation and differentiation, maintaining a stable internal environment of the cell, and accelerating wound tissue fibrosis [19]. It can increase the expression of type I/III collagen before chronic wounds in rats to promote wound healing [20]. Lindner [21] found that AKT1 is involved in the regulation of defensor expression and an important innate immune response to bacterial clearance, which is particularly important in the early control of inflammation in chronic infectious wounds. Huang [22] designed in vivo and in vitro models. Compared with the control group, skin damage caused the upregulation of TNF-*α* expression in the peripheral blood of diabetic rats and a significant increase in M1 macrophage infiltration, which proved that the high-glucose environment that was caused by single nuclear cells is more prone to the polarization of M1 type macrophages. Yue [23] proposed that c-Jun overexpression promotes the proliferation and migration of human umbilical cord-derived mesenchymal stem cells (hUC-MSCs) in vitro and accelerates diabetic wound healing, re-epithelialization, and angiogenesis through hUC-MSCs in vivo. The ESR1 and ESR2 genotypes are associated with insulin sensitivity and metabolic syndrome in Japanese and Chinese women. The ESR1 gene is important in preventing and reducing inflammation and glucose tolerance [24, 25]. In the pathogenesis of diabetic wounds, the expression level of MMP9 increases, impairing the balance of ECM synthesis, and degradation can cause the wound to be difficult to heal. In addition, the expression level of MMP9 gradually decreases as the wound heals [26, 27]. Therefore, the extract of *Periplaneta americana* may promote the healing of diabetic anal fistula wounds through multicomponent regulation and multitarget synergy.

GO enrichment analysis found that the extract of *Periplaneta americana* mainly promoted wound healing after diabetic anal fistula surgery through biological processes such as cell proliferation, macrophage differentiation, angiogenesis, and response to hypoxia. Studies have found that the macrophage subgroup Ly6CHi cells show a peak of MCP-1 in diabetic wounds. However, the Ly6CHi influx is reversed by the selective administration of anti-MCP-1, thus improving wound healing [28]. In vitro studies have shown [22] that a high glucose environment is conducive to the polarization of M1 type macrophages and produces a large amount of TNF-*α*, which is not conducive to the migration of keratinocytes and affects wound healing. Through the analysis of the KEGG pathway, it was found that the main enriched PI3K-Akt signaling pathway, HIF-1 signaling pathway, and estrogen signaling pathway may play an important role in diabetic anal fistula wounds. Epidemiological studies have shown that the occurrence of the anal fistula may be closely related to sex hormone levels and anal gland infection [29]. The genes enriched in the estrogen signaling pathway are JUN, MMP2, MAPK1, AKT1, ESR1, MMP9, and EGFR. Except for MMP2, the rest belong to the top 10 key genes in the PPI network. El-Tawil found that the rate of women suffering from congenital fistula is lower than that of men. Estrogen has anti-inflammatory activity and can antagonize the stimulation of the lipopolysaccharide component (LPS) of the cell wall of Gram-negative bacteria. Studies have confirmed that estrogen can accelerate wound healing, by reducing wound macrophage infiltration, inhibiting LPS-induced inflammatory signals, promoting the migration of mouse keratinocytes in vitro, promoting wound formation in diabetic mice, improving insulin sensitivity, and stimulating glucose to ingest [30–33]. Estrogen may become a new therapeutic target for diabetic anal fistula wounds. Clinical studies have found that, with the aggravation of male diabetic foot ulcers, the level of estradiol in the body increased and the level of testosterone decreased [34]. J. Xia et al. [35] found that the androgen level of anal fistula patients was higher than that of patients with nonanal fistula, which may be related to the rapid proliferation of the anal glands caused by the elevated testosterone level of patients. The role of sex hormones on the wounds of diabetic anal fistula surgery remains to be confirmed by further studies.

## 5. Conclusion

In summary, *Periplaneta americana* extracts may participate in biological processes such as macrophage differentiation, angiogenesis, and response to hypoxia. Regulation of PI3K-Akt signaling pathway, HIF-1 signaling pathway, and estrogen signaling pathway promotes wound healing. This study clarified the relationship between the main components, targets, pathways, and diseases of the American cockroach extract through network pharmacology techniques and approaches and elucidated that it can promote wound healing through multiple targets and multiple pathways. The above findings provide an important theoretical basis for further experimental research.

## Figures and Tables

**Table 1:** Topological parameters of main ingredients.

Node	Degree	Closeness	Betweenness
Cyclo-(L-Val-L-Pro)	24	0.45512821	0.4782475
(E)-3-Hexenyl-*β*-D-glucopyranoside	17	0.41764706	0.28230501
Arbutin	14	0.40804598	0.20036162
Uridine	12	0.3988764	0.06677689
Cytidine	12	0.39444444	0.05409036
Inosine	12	0.39444444	0.07432615
Guanosine	13	0.39444444	0.06911849
Adenine	9	0.38586957	0.11076596
Uracil	5	0.36979167	0.05474434

## Data Availability

The data used to support the findings of this study are included within the article.
